# Functional neural network analysis in frontotemporal dementia and Alzheimer's disease using EEG and graph theory

**DOI:** 10.1186/1471-2202-10-101

**Published:** 2009-08-21

**Authors:** Willem de Haan, Yolande AL Pijnenburg, Rob LM Strijers, Yolande van der Made, Wiesje M van der Flier, Philip Scheltens, Cornelis J Stam

**Affiliations:** 1 Alzheimer center and Department of Neurology, VU University Medical Center, Amsterdam, the Netherlands; 2 Department of Clinical Neurophysiology, VU University Medical Center, Amsterdam, the Netherlands

## Abstract

**Background:**

Although a large body of knowledge about both brain structure and function has been gathered over the last decades, we still have a poor understanding of their exact relationship. Graph theory provides a method to study the relation between network structure and function, and its application to neuroscientific data is an emerging research field. We investigated topological changes in large-scale functional brain networks in patients with Alzheimer's disease (AD) and frontotemporal lobar degeneration (FTLD) by means of graph theoretical analysis of resting-state EEG recordings. EEGs of 20 patients with mild to moderate AD, 15 FTLD patients, and 23 non-demented individuals were recorded in an eyes-closed resting-state. The synchronization likelihood (SL), a measure of functional connectivity, was calculated for each sensor pair in 0.5–4 Hz, 4–8 Hz, 8–10 Hz, 10–13 Hz, 13–30 Hz and 30–45 Hz frequency bands. The resulting connectivity matrices were converted to unweighted graphs, whose structure was characterized with several measures: mean clustering coefficient (local connectivity), characteristic path length (global connectivity) and degree correlation (network 'assortativity'). All results were normalized for network size and compared with random control networks.

**Results:**

In AD, the clustering coefficient decreased in the lower alpha and beta bands (p < 0.001), and the characteristic path length decreased in the lower alpha and gamma bands (p < 0.05) compared to controls. In FTLD no significant differences with controls were found in these measures. The degree correlation decreased in both alpha bands in AD compared to controls (p < 0.05), but increased in the FTLD lower alpha band compared with controls (p < 0.01).

**Conclusion:**

With decreasing local and global connectivity parameters, the large-scale functional brain network organization in AD deviates from the optimal 'small-world' network structure towards a more 'random' type. This is associated with less efficient information exchange between brain areas, supporting the disconnection hypothesis of AD. Surprisingly, FTLD patients show changes in the opposite direction, towards a (perhaps excessively) more 'ordered' network structure, possibly reflecting a different underlying pathophysiological process.

## Background

Understanding the relation between structure and function of the brain is one of the basic questions of neuroscience. Although a large body of knowledge about both healthy and pathological brain structure and function has been gathered over the last decades, we still have a poor understanding of their exact relationship. A clinical illustration of this state of affairs is dementia, a syndrome in which the link between pathophysiology and clinical symptoms is often ambiguous. There is a general consensus that cognition is a highly distributed and dynamic process, and thus depends on the coordinated interaction between many brain regions. It therefore seems reasonable to assert that approaches with an emphasis on structural damage will not be able fully explain cognitive (dys)function, since the complex interactions and interdependencies between different regions are neglected. A more complete perspective would have to take into account both the local and the global structural changes as well as the dynamics of the brain, and the way these different aspects are related. Therefore, several authors have argued that in addition to our present knowledge a more integrative network or system view on the brain is necessary [1-3]. Over the last decade, due to the development and interdisciplinary combination of techniques and methods, network analysis applied to biological research fields such as immunology, genetics and neuroscience has taken a great flight.

A novel approach, applying concepts from graph theory (a branch of the mathematical field of complex network theory) to neurophysiological data, is a promising new way to characterize brain activity [4-6]. It provides a method to evaluate whether the functional connectivity patterns between brain areas resemble the organization of theoretically efficient, flexible or robust networks (based on the strenght of synchronization in the oscillatory electromagnetic activity of different brain regions as measured by EEG or MEG). A fundamental hypothesis is that cognitive dysfunction can be illustrated and/or explained by a disturbed functional organization. Applied to patient data, this technique might provide more insight in the pathophysiological processes underlying the various forms of dementia, and potentially lead to the development of new diagnostic or monitoring tools.

Graph theory provides a method to study the relation between network structure and function, concerning for example qualities such as network efficiency, robustness, cost, or growth. Watts and Strogatz introduced so-called 'small-world' networks, that have an optimal balance between local specialization and global integration [7]. Small-world networks are optimal in the sense that they allow efficient information processing, are (wiring) cost-effective, and relatively resilient to network damage. Many real-life systems appear to have small-world properties [7-9]. Both anatomical and functional brain networks can be described by forming graphical network representations based on the measured (functional) connections. The presence of small-world network organization in brains of healthy humans was confirmed in numerous studies [5,6,10-14]. A few studies have recently shown that different types of brain pathology interfere with the normal small-world architecture [15-17]. Furthermore, a loss of small-world network properties in several frequency bands of EEG and MEG recordings in AD has been reported, with a more 'random' overall network structure [12,18]. Loss of small-world structure in AD was also demonstrated in recent MRI studies applying graph theory [19,20].

In view of these findings, one might speculate that other types of dementia also demonstrate a disturbance of the 'normal' small-world configuration of brain networks, perhaps even in a disease-specific way. This hypothesis is explored in the present study.

Many network characteristics can be used to examine neuroscientific data [5,6]. Since our current interest was mainly with (loss of) general structure, we expanded our analysis with a third measure, the so-called 'degree correlation' (R) [8,21]. It describes the tendency of nodes to form connections with nodes with similar degree. With a positive degree correlation, the chance that a node with a certain amount of connections neighbors other nodes with approximately the same amount of connections is larger. When this is the case for many nodes, a graph is called 'assortative'. Interestingly, most social networks tend to be assortative, while most biological networks tend to be disassortative. Assortative networks are thought to be better connected as a whole, and more robust to damage, i.e. deletion of connections [22].

In FTLD, a neurodegenerative disorder that is associated with more focal pathology in the frontal and/or temporal areas, we expected to find changes in functional network organization, but not identical to the situation in AD. The observation that many patients with a clinical manifestation of FTLD lack typical structural abnormalities on neuro-imaging suggests that functional changes might play a more important role [23]. We therefore set out to study functional networks both in patients with AD and FTLD. Subjects and EEG data were identical to Pijnenburg et al. [24].

## Results

### Subject characteristics

The main subject characteristics are summarized in Table 1. The FTLD group consisted of more males than the other two groups. Therefore, both SL and network measures were assessed for each gender group, not leading to any significant differences.

**Table 1:** Subject characteristics

	AD	FTLD	Controls	Significance
Age	65.5 (51–76)	63 (43–79)	59 (49–78)	p = 0.48
M:F	7:13	12:3	14:9	p = 0.02

MMSE	21.5 (14–27)	24.5 (13–30)	29 (27–30)	p = 0.09^1^ p < 0.001^2^ p = 0.002^3^

^1 ^= AD compared to FLTD, ^2 ^= AD compared to controls, ^3 ^= FTLD compared to controls. AD = Alzheimer's disease, FTLD = frontotemporal lobar degeneration, M = male, F = female

### Graph analysis

All subjects demonstrated small-world network properties in all frequency bands, expressed by the finding that the small-worldness (σ) values were larger than 1 in all frequency bands. In our study, the only significant change in σ was found in the AD group, where a decrease compared to controls was found in the beta band (p < 0.05).

**Figure 1:**
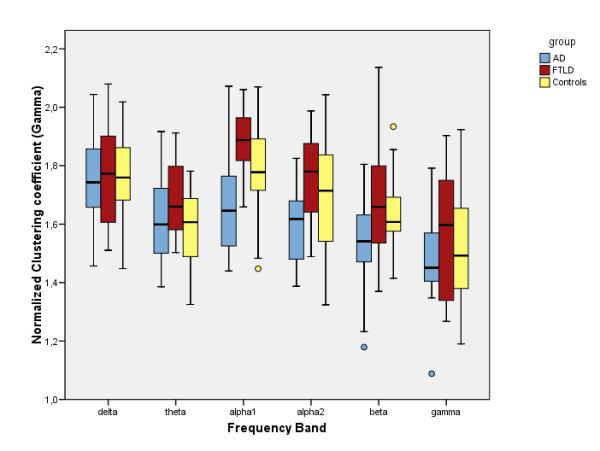
**Clustering coefficient**. Boxplots showing differences in normalized clustering coefficients (γ) for the separate frequency bands. Alpha1 = lower alpha band (8–10 Hz), alpha2 = upper alpha band (10–13 Hz).

**Figure 2:**
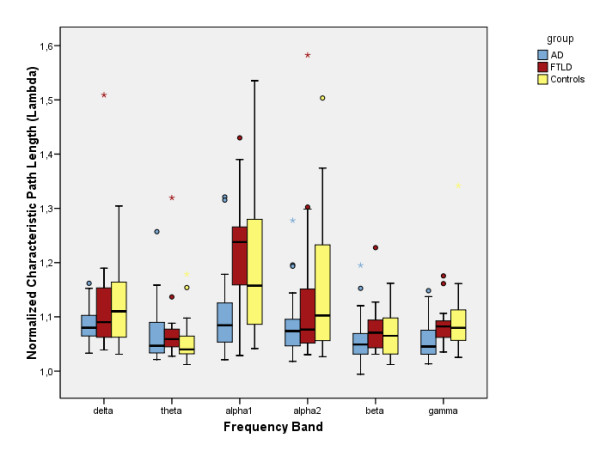
**Characteristic path length**. Boxplots showing differences in normalized characteristic path lengths (λ) for the separate frequency bands. Alpha1 = lower alpha band (8–10 Hz), alpha2 = upper alpha band (10–13 Hz).

**Figure 3:**
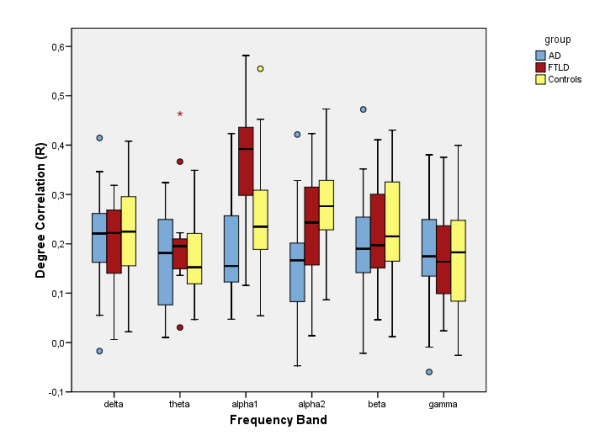
**Degree Correlation**. Boxplots showing differences in degree correlation (R) for the separate frequency bands. Alpha1 = lower alpha band (8–10 Hz), alpha2 = upper alpha band (10–13 Hz).

Clustering coefficient, characteristic path length and degree correlation results have been summarized in figures 1, 2 and 3. The mean, normalized clustering coefficient (γ) was decreased in AD compared to controls in the lower alpha (p < 0.05) and beta (p < 0.05) frequency bands. FTLD showed a non-significant but constant trend in opposite direction in the higher frequency bands. In all frequency bands, AD and FTLD median values changed in opposite directions, reaching significance in both alpha bands (p < 0.01).

The normalized characteristic path length (λ) was decreased in AD compared to controls in the lower alpha (p < 0.05) and gamma (p < 0.01) frequency bands. In those same bands, the difference between AD and FTLD was highly significant (p < 0.01 and p < 0.05, respectively). In the FTLD group, no differences with the control group were found.

The degree correlation (R) was decreased in AD compared to controls and FTLD in both alpha frequency bands (p < 0.05 and p < 0.01, respectively). In FTLD compared to controls, the *increase *in R was highly significant in the lower alpha band (p < 0.01).

All normalized network measures were within the same range as previously reported results (see also table 2).

**Table 2:** Comparison of small-world characteristics with AD network literature

Study	group	N	γ	λ	σ
Present study	Control group	21	1.67	1.11	1.50
EEG (Stam 2007)	Healthy controls	21	1.58	1.07	1.48
MEG (Stam 2004)	Healthy controls	126	4.20	1.80	2.30
fMRI (Supekar 2008)	Healthy controls	90	1.74	1.05	1.66
Present study	AD	21	1.61	1.08	1.49
EEG (Stam 2007)	AD	21	1.60	1.12	1.43
fMRI (Supekar 2008)	AD	90	1.56	1.04	1.50

Present study	FTLD	21	1.73	1.12	1.55

Comparison for unweighted network characteristics of the present study with earlier reported work. Although there are considerable methodological differences between these studies, all results indicate that network structure in both healthy persons and AD patients can be described as having small-world network characteristics (σ>1). N = number of nodes in the graph, γ = normalized clustering coefficient, λ = normalized characteristic path length, σ = small-worldness (γ/λ). In the EEG and MEG studies, values have been averaged over all frequency bands.

### Correlation between AD network characteristics and MMSE score

In the AD group, MMSE score was documented in 21 out of 23 subjects. In the lower alpha band in AD, normalized characteristic path length (λ) was positively correlated with MMSE score (r = 0.50, p < 0.05).

## Discussion

In this study we applied graph analysis to resting-state EEG data of AD, FTLD and control subjects to characterize the large-scale organization of brain networks based on functional connectivity strength. The main finding is that this approach is able to demonstrate notable differences in functional brain network organization in AD and FTLD patient groups. FTLD network changes were often significantly different and in opposite direction compared to AD, possibly reflecting a different underlying disease mechanism.

### Frontotemporal lobar degeneration

To the best of our knowledge, this is the first documentation of graph analysis applied to FTLD patient data. First, it is important to recognize that network characteristics can show change regardless of the fact that no significant changes in underlying functional connectivity were found [24]. This is because they should primarily reflect global (changes in) network organization, and not in connectivity strength. Although we found no significant changes in the clustering coefficient and characteristic path length in FTLD compared to controls, a consistent trend (especially in the higher frequency bands) was that these network variables *in*creased, and thus changed in the opposite direction compared to the AD group (see figures 1 and 2), leading to highly significant differences between FTLD and AD in the lower alpha frequency band. In a spectral analysis study, a similar divergence between AD and FTLD qEEG data was reported [25].

Clustering coefficient and path length are not the only graph measures sensitive to detect network structure. The degree correlation R increased significantly in the lower alpha band, which is also a sign of more structure in the network. The fact that only the degree correlation reached significance suggests that, in this case, it is a more sensitive measure for capturing network structure differences between FTD and AD. The tendency towards a more regular network structure can be interpreted as a deviation from the presumably optimally balanced small-world network architecture. Why this strong increase in degree correlation is mainly found in the lower alpha band is not easy to explain in physiological terms, but involvement of the alpha band in FTD has been reported before [26]

Since there are not many discriminating EEG measures between FTLD and healthy persons, the increased assortativity as measured by the degree correlation (R) in the FTLD lower alpha frequency band is intriguing. An assortative network is generally associated with a more efficient information processing and a lower vulnerability to network damage [8,21,22]. Thus, the higher degree correlation we found in FTD compared to healthy controls seems paradoxical. In this regard, it is interesting to note that hierarchy in a network has been described as the tendency of hubs to connect to nodes that are not otherwise connected to each other [27]. Assortativity and hierarchy might thus be reflected upon as complementary network phenomena. Basset et al. showed in their resting-state fMRI study that in the multimodal sub-network of persons with schizophrenia, assortativity increased and hierarchy decreased [28]. Our increase in assortativity in the FTLD lower alpha band could perhaps also be interpreted as a loss of network hierarchy in this regard.

Since the application of graph analysis to neuroscientific data is still a very new approach, it is too soon to relate FTD network analysis outcomes to FTD pathophysiology, and draw firm conclusions. However, based on recent literature, one could argue as follows: FTLD is usually characterized by frontotemporal dysfunction and/or atrophy and related neuropsychological impairments, like loss of executive functions. Seeley et al. recently demonstrated in an fMRI study that specific patterns of atrophy and functional network activity converge in several neurodegenerative diseases, including FTD [29]. Meunier et al. showed that human functional brain networks appear to be modular, and that a large frontal module has extensive connections with other brain areas [30]. In FTLD, particularly the fronto-subcortical and temporo-subcortical circuits are affected, whereas the parietal and occipital cortices are relatively spared. The frontal and temporal lobes are responsible for highly complex cognitive functions such as social cognition. Clinically, the disorder presents with personality and behavioral changes resulting for example in mental rigidity, loss of cognitive flexibility and perseveration. It is conceivable that FTLD leads to a pathologically ordered and rigid network by altering long-distance network traffic to and from the coordinating frontal areas, but this hypothesis has to be explored in future studies. Interestingly, in an fMRI study of ADHD patients a similar shift towards a more ordered network type was reported [31], and the same seems to be happening in patients with Parkinson's disease dementia (Olde Dubbelink KTE, unpublished results).

### Alzheimer's Disease

With decreasing local and global network parameters in AD in the present study, the large-scale functional brain network structure deviates towards a more random type. The loss of structure as expressed by the lower clustering and path length in the higher frequency bands in AD seems to support the notion of AD as a disconnection syndrome, together with the well known slowing of brain activity and loss of functional connectivity in AD [32]. The lower alpha band in particular has been related to global arousal/attention, and deterioration of this cognitive domain is a common feature of AD. The finding that the lower alpha band produces the most striking differences between AD and controls could suggest that network changes mainly affect the level of attention/arousal, which has an effect on other cognitive abilities, and thus contributes to the multi-domain, non-specific cognitive impairment as seen in AD. However, as work by Klimesch et al. has pointed out, attributing global arousal level as physiological meaning to the alpha band is more reliable when the individual alpha frequency peak (IAF) is taken into account [33]. For easier comparison with previous research, fixed bands were used in this study.

A recent magnetoencephalographic AD study showed very similar results: a decreased clustering coefficient and characteristic path length in the lower alpha band [18]. At first, a shorter path length related to a worse cognitive status seems counter-intuitive. However, theoretically, a shorter path length is not necessarily an advantage in a complex network, since it is the overall structure that must be an effective balance between local specialization and global integration. Decreases in both clustering coefficient and path length mean a more rapid shift towards network randomness. Earlier EEG work [12] did find an *in*crease of the characteristic path length in the beta frequency band in AD, not in line with the present findings. The explanation for this might be found in two methodological developments. First, for the present study (and the MEG study) a different algorithm was adopted for determining the characteristic path length, which deals better with disconnected nodes in a graph [22]. Another major difference is that here, the network measures are normalized by comparing them to random networks (see methods section for a more detailed explanation of both issues). For comparison with results from other studies, table 2 provides an overview of all AD-related graph analysis findings so far.

Our finding that in AD the R decreases in both alpha bands is in agreement with the notion of the AD network losing structure and becoming more random and disorganized, as shown by the decrease of γ and λ in AD. All these findings taken together seem to support the 'disconnection syndrome' hypothesis of AD; deterioration of cognition due to loss of functional connectivity and organization. The positive correlation of the characteristic path length with MMSE score in the lower alpha band in AD also supports this idea.

### Methodological issues

In this study a few issues regarding methodological limits or possible confounders should be addressed. Subjects and EEG data were identical to Pijnenburg et al., and several study limits have been discussed there [24].

Using SMC as a control group is a debatable choice, since people in this group have been reported to show differences compared to persons without SMC [34], and have a higher chance of having an underlying neurodegenerative disease such as AD or FTLD than healthy controls [35], and this might have led to a slight underestimation of group differences in our study. However, the chance that SMC subjects have an underlying FTLD is very small, and since FTLD and AD subjects showed opposite network changes, an underestimation of the differences between SMC and FTLD is not very likely. We have the following reasons for choosing SMC as a control group: First, SMC subjects are more representative of the population visiting memory clinics than completely healthy persons. Therefore, when searching for clinically relevant features, a comparison involving SMC might be more useful. Second, SMC subjects in our clinic have had a comprehensive screening with proven test methods, after which no objective impairments are found. The absence of cognitive impairment in this group might be more reliable then in a so-called 'healthy' control group who have not participated in extensive testing.

Another concern is medication use, since it can affect recorded brain activity [36,37]. However, since the EEG, MMSE and other diagnostic tests had been performed as part of the diagnostic process, no pharmacological therapy (like e.g. cholinesterase-inhibitors in AD) had yet been initiated. There was an incidental report on the use of pre-diagnostic psycho-active medication (benzodiazepine use in two FTD patients and two controls, Exelon use in two AD patients), but since these persons did not show outlying SL values, network analysis results or clinical characteristics, we are convinced this can not have had any notable influence on the results in this study.

While interpreting our results, readers should be aware of several statistical limitations: first, we did not apply corrections for multiple testing. However, since network measure data did not show a Gaussian distribution, we used nonparametric statistical testing, which makes less a priori assumptions. Also, the most important significant findings we report are not near the p = 0.05 level, and almost all the non-significant results in other bands showed constant trends in the same direction (see figures 1, 2 and 3), rendering it unlikely that significant effects are based on coincidence. Finally, in the non-parametric Kruskal-Wallis it is not possible to adjust for covariates such as age, but since our groups were age-matched this should not have a large effect.

A graph theoretical concern deals with the decision to form unweighted graphs based on binary connectivity matrices obtained by filtering the original SL values with an arbitrarily chosen threshold. A justified question is how to determine the height of this threshold, since network results are dependent on this. This question has been addressed in [12], where network variables were analyzed as a function of different K (mean degree of the network) thresholds. This was also to ensure that the resulting networks would be of similar size, and therefore more easily comparable in terms of structure. In a similar way we have analyzed network results across a range of K-values [see additional files 1, 2, 3 and 4]. To avoid disconnected and fully connected, random graphs, K values outside this range were not examined. For clarity reasons, we chose one threshold value (K = 5) as representative for the whole range. An alternative approach is to convert the original SL-based connectivity matrix directly into a 'weighted' graph, in which the connections between nodes in a graph have variable strengths. This approach is explored in a recent MEG-study in Alzheimer patients [18].

### Future directions

Whether functional network changes in neurophysiologic data can be linked to specific pathophysiological mechanisms or clinical symptoms, is still unclear at this stage, and further systematic study is needed. Graph theory offers a growing amount of techniques to describe topological network features like modularity, node centrality (e.g. 'betweenness'), or synchronizability [5,6,8,22]. Furthermore, comparison of network findings with other neurodegenerative diseases (e.g. Huntington's disease, PSP) and clinical/pathophysiological measures (e.g. (f)MRI, CSF, APOE status [38,39] or neuropsychological test-scores) would be of interest; it is conceivable that different cognitive symptoms arise from different types of network disturbance, or that neuronal or synaptic loss in discrete regions leads to specific network disturbance. Another relevant question is whether loss of neurotransmitter function (e.g. acetylcholine in AD) has notable implications for network function, because this could lead to a non-invasive method to monitor or even predict cholinergic status and potential medication effectiveness. Cholinergic effects have been associated with enhanced functional connectivity [36]. Finally, it would be of interest to compare graph analysis results of EEG and MEG recordings in the same individuals, and to look at longitudinal measurements, taking into account effects of aging and disease course.

## Conclusion

AD and FTLD patients show dissimilar resting-state functional brain network disturbance. Whereas in AD there is a general loss of connectivity and network structure, FTLD shows a tendency towards a more ordered network structure. This suggests that the approach used in our study, applying graph analysis to EEG data, can be used for identifying differences and possibly for gaining more insight in the pathophysiological processes underlying these forms of dementia. With this new, integrative perspective on large-scale brain function emerging, we may contribute to bridging the gap in our understanding between brain structure and function.

## Methods

### Patient diagnosis and recruitment

Subjects and EEG data were identical to Pijnenburg et al. [24]. Fifteen consecutive patients with FTLD according to the criteria of Neary and Snowden [40] were recruited from the Alzheimer Centre of the VU University Medical Centre. Twenty patients with probable AD according to the NINCDS-ADRDA criteria [41] matched for age and disease severity were drawn from the Alzheimer Center clinical database. All patients underwent a standard battery of examinations including medical history, informant-based history, physical and neurological examination, screening laboratory tests, psychometric tests, MRI, and EEG. All diagnoses were made by consensus in a multidisciplinary team. The diagnoses were kept under review and only considered correct if the clinical course over a period of at least one year of follow up was consistent with the diagnosis. Twenty-three subjects with subjective cognitive complaints served as a control group. They presented with cognitive (mostly memory related) complaints at our clinic, but were found to have no objective cognitive disorder after thorough testing (the same diagnostic procedure as described above). The study was conducted in accordance with regional research regulations and conformed to the Declaration of Helsinki.

### EEG Acquisition

EEGs were recorded using an OSG digital EEG equipment (Brainlab (R)) at the following positions of the international 10–20 system: Fp2, Fp1, F8, F7, F4, F3, A2, A1, T4, T3, C4, C3, T6, T5, P4, P3, O2, O1, Fz, Cz, Pz with an average reference (including all electrodes except Fp2/1 and A2/1). ECG was recorded in a separate channel. Electrode impedance was below 5 kOhm. Initial filter settings were: High pass filter = 0.16 Hz, low pass filter = 70 Hz. Sample frequency was 500 Hz and A-D precision 16 bit. Subjects were seated in a slightly reclined chair in a sound attenuated, dimly lit room, and instructed to stay alert as much as possible during the whole recording. Further offline post-processing and epoch selection was performed by an experienced investigator (CS), who was blinded to the diagnosis, and who took care to exclude data with artifacts due to for example (eye) movements, drowsiness, or technical issues. For this study, 4 epochs (sample frequency 500 Hz; 8.19 s) of a no-task eyes-closed condition were selected and band-pass filtered for the commonly used frequency bands: delta (0,5–4 Hz), theta (4–8 Hz), lower alpha (8–10 Hz), upper alpha (10–13 Hz), beta (13–30 Hz) and gamma (30–45 Hz). All further analyses were performed for these bands separately.

### Graph theory

**Figure 4:**
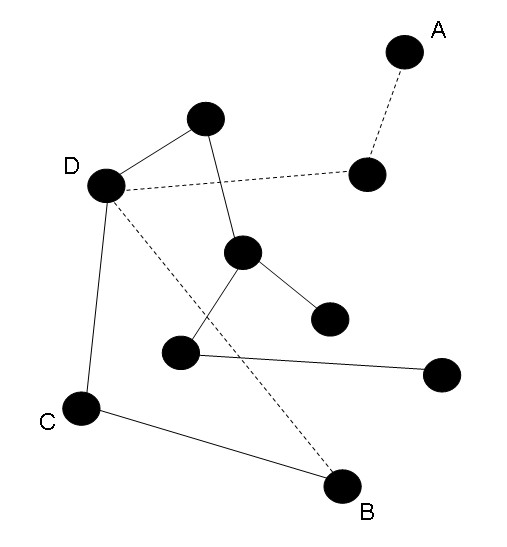
**Graph theory principles**. Graphs can represent any kind of network. Dots represent *nodes*, and lines connecting the dots are the *connections*. The **degree (K) **of a node is it's number of connections. The **clustering coefficient (C)**, measuring local connectivity of a node, is the likelihood that its neighbors are connected. For node C, with neighbours B and D, the clustering coefficient is 1. The **characteristic path length (L)**, a measure of global connectivity, is the minimum number of connections between two nodes. The path length between vertices A and B consists of three edges, indicted by the striped lines. The **degree correlation (R)**, a measure of network clustering according to degree, is the ratio of the degrees of two neighboring nodes. Figure taken with permission from *Stam and Reijneveld. Graph theoretical analysis of complex networks in the brain. Nonlinear Biomedical Physics. 2007c; 1: 3*.

A short illustration of the basic principles of graph theory used in this study is provided in figure 4.

**Figure 5:**
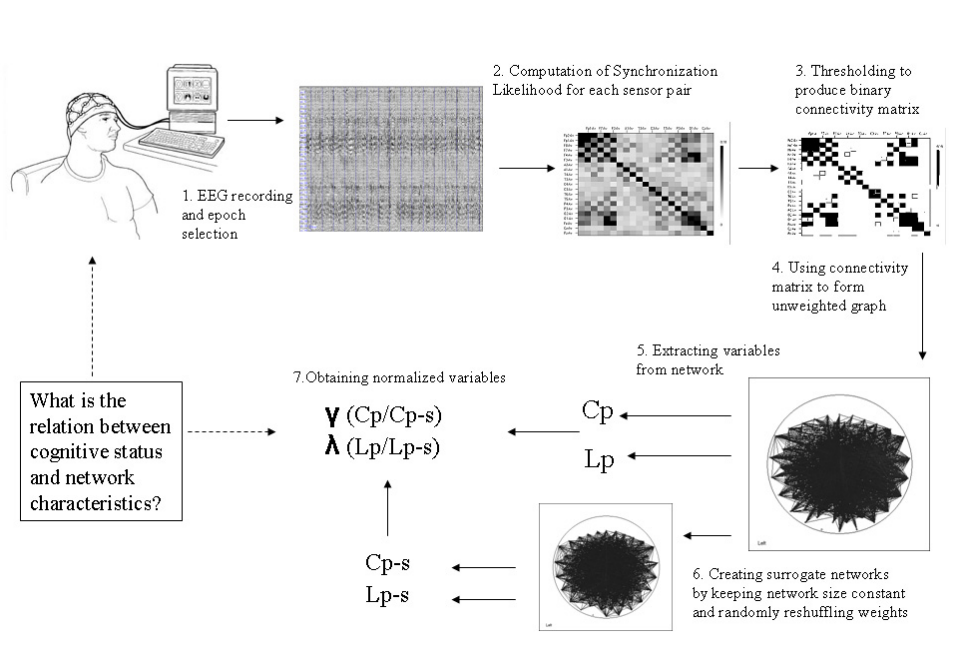
**From EEG recording to unweighted graph**. Multi-step procedure to obtain normalized network-derived variables. C = clustering coefficient, L = Characteristic path length, γ = normalized clustering coefficient, λ = normalized characteristic path length.

Graphical representations of the functional brain network are formed using the functional connectivity measure 'synchronization likelihood'(SL) as a basis; this multi-step procedure is outlined in figure 5. The SL is a general measure of the synchronization between two time series, sensitive for linear and nonlinear interdependencies. SL procedure and results for this group have been published in [24]. A more detailed technical description is provided in [42,43].

**Figure 6:**
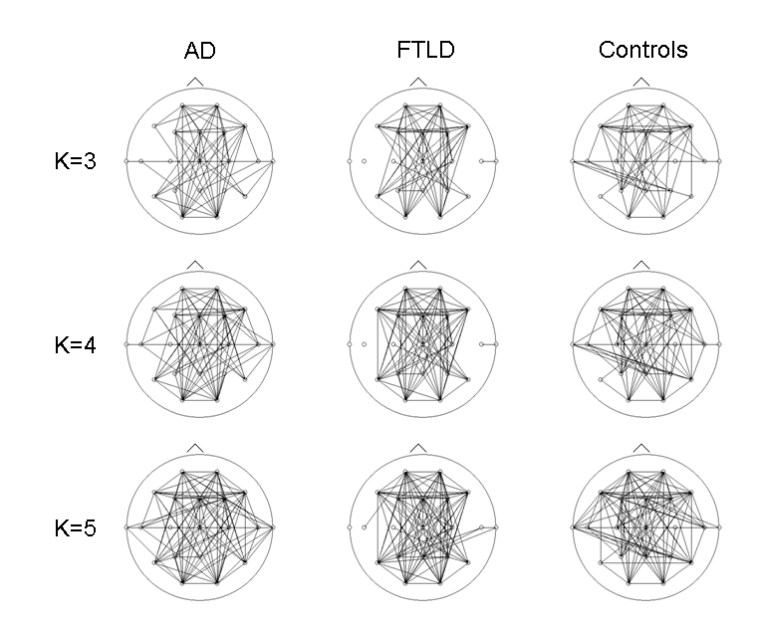
**Unweighted graphs of the lower alpha band (8–10 Hz) for different patient groups and different fixed average degrees (K)**. For the AD, FTLD and control groups, the functional connectivity (SL) based graphs are shown as headplots for different values of K. Lower K values (higher threshold) result in a sparser network. On visual inspection, it is obvious that there are inter-group differences.

For each frequency band, the SL calculation produces a value of connectivity strength for every sensor pair, which results in a matrix showing the connectivity between all possible sensor pairs (step 2 in figure 5). For this study we used unweighted, binary graphs, which means that only connections with a SL value higher than a (chosen) threshold will be realized in the representing network graph. Here, an important methodological problem has to be tackled; when forming graphs (step 3 in figure 5), the results might be influenced by differences in the mean level of synchronization between groups. Because the SL is expected to be significantly lower for Alzheimer patients than controls, for a given threshold, AD graphs will have fewer connections than the controls graphs. Therefore, thresholds are chosen in such a way that the resulting graphs of the different groups have an equal mean degree K (see figure 6). Persisting dissimilarities between group networks will more likely be due to true differences in network organization.

Since network-derived measures are not just dependent on network structure, but also on network size, between-group comparison should be done on networks of equal size. To achieve this, the SL-threshold is chosen in such a way that graphs in both groups are guaranteed to have the same average number of edges, so that any remaining network differences between the groups reflect differences in graph structure. Because the choice of the threshold is arbitrary, a range of different thresholds is examined (see Additional files 1, 2, 3 and 4).

Graphs can be formed by a set of nodes and connections, and can then be characterized by various measures (step 4 and 5 in figure 5). The number of connections a node possesses is called the *degree *(k) of that node. The degree (K) of a network is the average degree of all nodes. In the following analyses the results of networks with an average degree of K = 5 are shown, since they were representative for the findings at other threshold levels. Two other core network measures are the *clustering coefficient *C and the *characteristic path length *L (see also figure 4). The clustering coefficient C of a node is the ratio of all existing connections between the 'neighbors' of a node (nodes that are one step away) and the maximum possible number of edges between the neighbors. The mean clustering coefficient is computed for all nodes of the graph and then averaged. It is a measure for the tendency of network elements to form local clusters. The characteristic path length is the average shortest path connecting any 2 nodes of the graph: the length of a path is indicated by the number of connections it contains. The characteristic path length L (averaged shortest path length between all node pairs) is an emergent property of the graph, which indicates how well its elements are integrated/interconnected. In the conventional method to calculate path length L, disconnected nodes in a network pose a problem. Newman proposed to define L to be the 'harmonic mean' distance between pairs, or the reciprocal of the average of the reciprocals [22]. In this way, calculation of L resembles the 'global efficiency' introduced by Latora and Marchiori [44].

To obtain normalized measures, network-derived variables are compared with 50 control networks of the same size (step 6 and 7 in figure 5). In the resulting connectivity matrices (after SL computation), all sensor values were consecutively swapped with a different sensor value in the same diagonal halve of the matrix. Since the networks are undirected, both diagonals should be symmetrical, and therefore the new, 'swapped' halve was copied to the other halve of the matrix. This results in an equally-sized network with an identical degree distribution, but a different structure. This same procedure was repeated to obtain 50 random surrogate networks. Gamma (γ) is used for the normalized C (C/C-random), and Lambda (λ) is used for the normalized L (L/L-random).

'Small-worldness' (σ) is the ratio of γ and λ, and is used to describe the balance between the local connectedness and the global integration of a network. When this ratio is larger than 1, a network is said to have Small-world properties [9].

Another investigated graph property concerning network structure is the degree correlation [8,21,22]. The degree correlation (R) indicates whether the degree of a node is influenced by the degree of another node to which it connects. The degree correlation is calculated by obtaining the Pearson's correlation of the degrees of two connected nodes, repeating this for every connected node pair, and then averaging these correlations.

Correlations between network measures and MMSE score were tested for the AD group only, since documentation for the FTD group was incomplete (10 of 15 MMSE scores known).

### Statistical evaluation

For statistical analysis, the SPSS 15.0 package for Windows was used. Since not all network-derived variables showed a Gaussian distribution (Kolmogorov-Smirnov test), network variable comparison between the three diagnostic groups was performed using nonparametric statistics (Kruskal-Wallis test followed by Mann Whitney-U tests when appropriate). Correlations between network measures and MMSE score were calculated with Spearman's correlations. Separate analyses were performed for each of the six frequency bands. A significance level of α = 0.05 was used.

## Authors' contributions

WH performed all analyses, and wrote most of the manuscript. CS designed the study, gave advise on neurophysiological and graph theoretical issues, and helped to draft the manuscript. YP recruited and examined patients, gave advise on clinical issues, and helped to draft the manuscript. WF gave advise on statistical and methodological issues, and helped to draft the manuscript. PS, RS and YM helped to draft the manuscript. All authors read and approved the final manuscript.

## Supplementary Material

Additional file 1 **Threshold analysis in the lower alpha frequency band (8–10 Hz)**. Graph analysis results of unweighted networks as presented in this paper are dependent on an arbitrarily chosen threshold (in our study K, mean degree of the network). This supplement, including 3 figures, shows that the reported results (K = 5) are representative for a broad range of K thresholds. Click here for file

Additional file 2 **Clustering coefficient**. Group comparison of the normalized clustering coefficient (Cp/Cp-s or γ) between conditions for different mean network degrees K (* p < 0.05 ** p < 0.01 compared to SMC). Click here for file

Additional file 3 **Path Length**. Group comparison of the normalized characteristic path length (Lp/Lp-s or λ) between conditions for different mean network degrees K (* p < 0.05 ** p < 0.01 compared to SMC). Click here for file

Additional file 4 **Degree correlation**. Group comparison of the degree correlation (R) for different mean network degrees K (* p < 0.05 ** p < 0.01 compared to SMC). Click here for file
