# Supplementary material for: Functional neural network analysis in frontotemporal dementia and Alzheimer's disease using EEG and graph theory
Source: BMC Neurosci. 2009 Aug 21;10:101. doi: 10.1186/1471-2202-10-101 (PMC2736175; doi:10.1186/1471-2202-10-101)
Supplement: Additional file 1 — Threshold analysis in the lower alpha frequency band (8–10 Hz). Graph analysis results of unweighted networks as presented in this paper are dependent on an arbitrarily chosen threshold (in our study K, mean degree of the network). This supplement, including 3 figures, shows that the reported results (K = 5) are representative for a broad range of K thresholds. [file 1471-2202-10-101-S1.doc]

**Additional file 1 – Threshold analysis**

Graph analysis results of unweighted networks as presented in this paper are dependent on an arbitrarily chosen threshold. To ensure reliability of findings, additional comparative analysis of network metrics as a function of threshold was performed (see for comparison [12]). Because most striking differences between patient groups were found in the lower alpha band, we focus on this frequency band.

For easier comparison between groups, we chose thresholds in such a way that the mean degree K was the same in the resulting networks. A K value is also more informative about a network then the real threshold value, since the latter depends on the connectivity measure used. Therefore, the figures of this threshold analysis report K-values on the X-axis, instead of the true threshold value.

The threshold analysis shows that the presented results are a robust finding, and the figures below illustrate that the significant results we found in the reported K=5 networks were representative for a broader range. Since small-world structure is best studied in sparse networks, and networks with an increasing mean degree will eventually become ‘random’ by definition, higher K levels than reported in the following figures were not studied. Likewise, to avoid disconnected graphs, lower K levels were not studied. [see additional files 2,3,and 4]

See also subsection ‘Methodological issues’ in the discussion section.
