# Supplementary figures and images for: Functional neural network analysis in frontotemporal dementia and Alzheimer's disease using EEG and graph theory
Source: BMC Neurosci. 2009 Aug 21;10:101. doi: 10.1186/1471-2202-10-101 (PMC2736175; doi:10.1186/1471-2202-10-101)

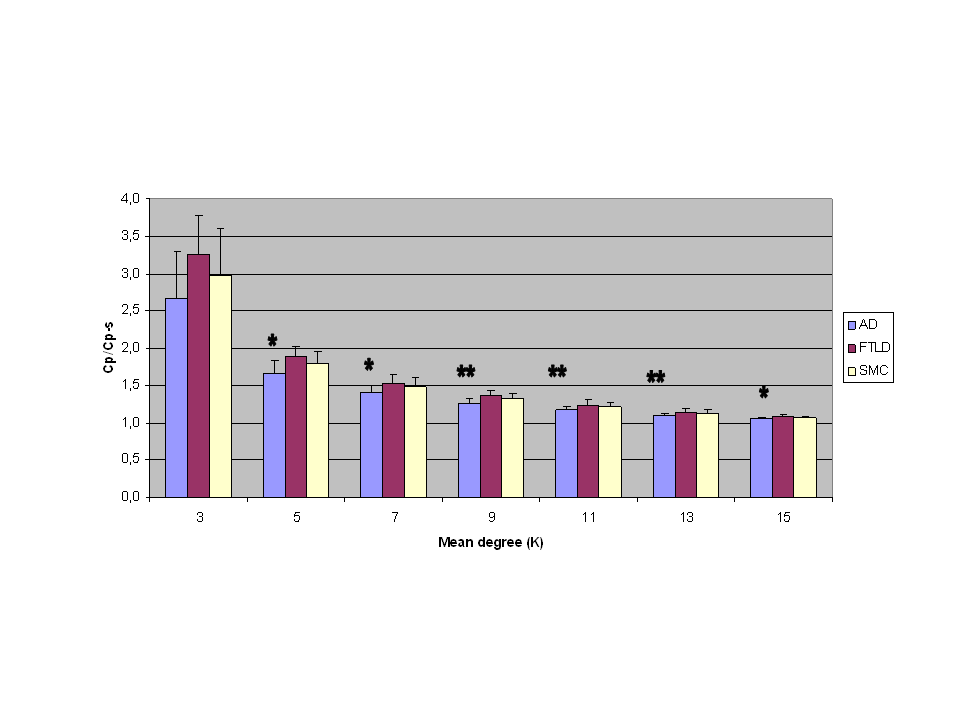

Supplement: Additional file 2 — Clustering coefficient. Group comparison of the normalized clustering coefficient (Cp/Cp-s or γ) between conditions for different mean network degrees K (* p < 0.05 ** p < 0.01 compared to SMC). [file 1471-2202-10-101-S2.tiff]

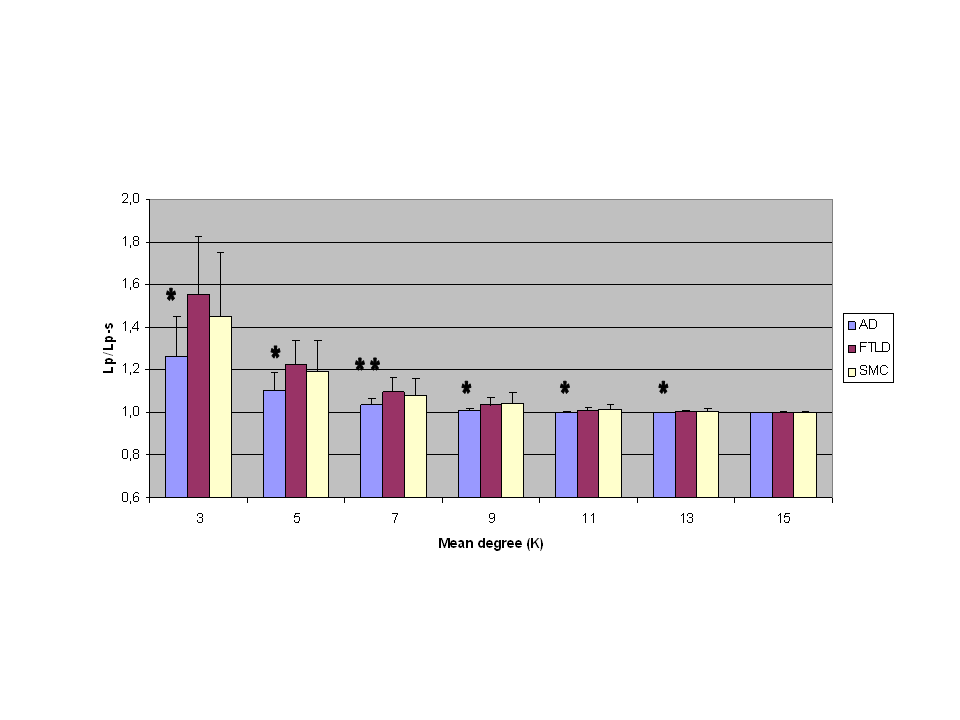

Supplement: Additional file 3 — Path Length. Group comparison of the normalized characteristic path length (Lp/Lp-s or λ) between conditions for different mean network degrees K (* p < 0.05 ** p < 0.01 compared to SMC). [file 1471-2202-10-101-S3.tiff]

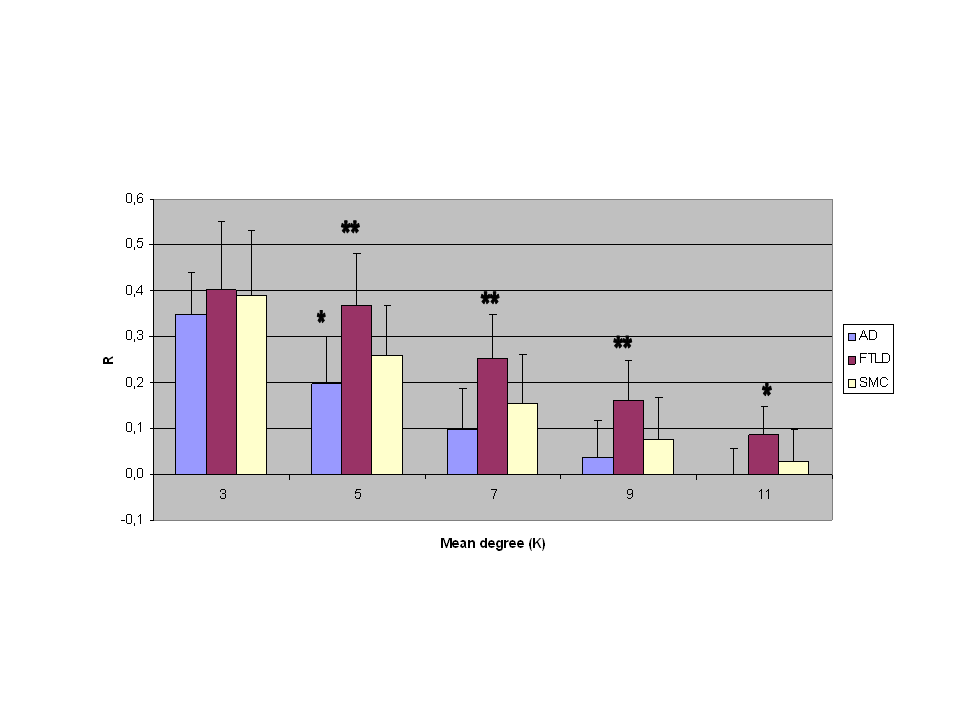

Supplement: Additional file 4 — Degree correlation. Group comparison of the degree correlation (R) for different mean network degrees K (* p < 0.05 ** p < 0.01 compared to SMC). [file 1471-2202-10-101-S4.tiff]
